# Pneumococcal conjugate vaccines and antimicrobial resistance: serotype replacement, clonal dynamics, and future vaccine perspectives

**DOI:** 10.3389/fimmu.2026.1903459

**Published:** 2026-07-24

**Authors:** Ziyi Yan, Yingying Li, Li Liu, Yanying Ren, Wenjing Wu, Liting Liang, Xingxin Liu, Wei Zhou, Linghan Kuang, Yali Cui, Yongmei Jiang

**Affiliations:** 1 Department of Laboratory Medicine, West China Second University Hospital, Sichuan University, Chengdu, China; 2 Key Laboratory of Birth Defects and Related Diseases of Women and Children of Ministry of Education, West China Second University Hospital, Sichuan University, Chengdu, China; 3 Department of Laboratory Medicine, Women and Children’s Hospital of Tibet Autonomous Region, Lhasa, China; 4 Department of Blood Transfusion, Beijing Anzhen Nanchong Hospital of Capital Medical University & Nanchong Central Hospital, Nanchong, China; 5 Department of Medical Laboratory, Dazhou Integrated Traditional Chinese Medicine & Western Medicine Hospital, Dazhou Second People’s Hospital, Dazhou, China; 6 Department of Laboratory Medicine, Chengdu Hi-Tech Zone Hospital for Women and Children (Chengdu Hi-Tech Zone Hospital for Maternal and Child Healthcare), Chengdu, China; 7 Department of Laboratory Medicine, West China Second University Hospital (Tianfu), Sichuan University (Sichuan Provincial Children’s Hospital), Meishan, China; 8 Department of Laboratory Medicine, Meishan Women and Children’s Hospital, Alliance Hospital of West China Second University Hospital, Sichuan University, Meishan, China

**Keywords:** antimicrobial resistance, clonal dynamics, global pneumococcal sequence cluster, pneumococcal conjugate vaccine, serotype replacement, *Streptococcus pneumoniae*, trained immunity

## Abstract

Pneumococcal conjugate vaccines (PCVs) have transformed the epidemiology of pneumococcal disease while providing a real-world model for understanding how vaccination reshapes antimicrobial resistance (AMR) in *Streptococcus pneumoniae*. In this Mini Review, we examine PCV-associated AMR dynamics through linked epidemiological, ecological, and genomic processes. We first distinguish two complementary AMR-reducing pathways: direct suppression of vaccine-type serotypes that historically carried antibiotic-nonsusceptible lineages, and indirect reduction of antibiotic exposure by preventing pneumococcal and respiratory disease syndromes that commonly trigger empirical treatment. We then explain why these effects are incomplete. The capsular biosynthesis operon, antimicrobial resistance determinants, and virulence protein genes occupy distinct genomic layers; therefore, post-PCV AMR outcomes depend not only on serotype removal, but also on capsular switching, recombination, mobile resistance elements, lineage fitness, carriage reservoirs, and antibiotic selection. Recent whole-genome sequencing and Global Pneumococcal Sequence Cluster studies show that serotype replacement is best interpreted as lineage-level ecological restructuring, with region-specific consequences for resistant disease. We further discuss how global and regional epidemiology, including serotype-specific invasiveness and uneven vaccine uptake, modifies PCV-associated AMR impact. Finally, we consider higher-valency PCVs, protein-based vaccines, and trained-immunity-based host-directed strategies as complementary future directions. We argue that next-generation pneumococcal vaccines should be evaluated not only by immunogenicity and serotype coverage, but also by their effects on antibiotic use, carriage dynamics, resistant lineage expansion, capsular switching, and long-term population-genomic outcomes.

## 1 Introduction

Antimicrobial resistance (AMR) is a major threat to the treatment of bacterial infections, and vaccination is increasingly recognized as an upstream intervention that can reduce infection burden, antibiotic exposure, and the transmission of resistant pathogens. *Streptococcus pneumoniae* is particularly informative in this context because it is a major cause of respiratory and invasive disease, a frequent driver of antibiotic prescription, and a pathogen in which resistance is closely linked to the population structure of circulating serotypes and clones (1–3).

Pneumococcal conjugate vaccines (PCVs) have provided one of the clearest examples of how vaccination can influence AMR at the population level. PCVs do not target resistance genes directly; rather, they reduce disease and carriage caused by vaccine-type pneumococci, including serotypes and lineages historically associated with antibiotic nonsusceptibility, while also reducing some pneumococcal and respiratory disease syndromes that commonly lead to empirical antibiotic use (1–3). However, the long-term effect of PCVs is not simply a one-directional decline in resistance. Global evidence has shown that 10-valent and 13-valent PCVs (PCV10 and PCV13) substantially reduced invasive pneumococcal disease, while non-vaccine serotypes increased over time after vaccine introduction (2, 4). This makes pneumococcal vaccination a useful model for examining how vaccine pressure, antibiotic selection, serotype replacement, clonal dynamics, and regional epidemiology jointly reshape AMR.

This Mini Review summarizes how PCVs have modified pneumococcal AMR through direct reduction of resistant vaccine-type serotype reservoirs, indirect reduction of antibiotic exposure, serotype replacement, and lineage-level ecological restructuring. It further discusses the genomic separation between the capsule locus, AMR determinants, and dispersed protein vaccine targets, and considers the implications of global and regional epidemiology for higher-valency PCVs and future vaccine strategies. Finally, we discuss serotype-independent protein vaccines and host-directed approaches such as trained-immunity-based vaccines as complementary directions, using the PCV experience as a real-world model for evaluating next-generation vaccines by their effects on antibiotic exposure, resistant lineage expansion, carriage ecology, and long-term pneumococcal population structure (5, 6).

## 2 Mechanisms by which PCVs reduce pneumococcal AMR

PCVs can reduce pneumococcal AMR through two complementary mechanisms. Here, “direct” refers to a population-level effect on vaccine-targeted resistant pneumococci, rather than direct molecular targeting of resistance genes. The first mechanism is therefore a serotype-mediated direct population effect: PCVs target capsular serotypes that historically contained major antibiotic-nonsusceptible pneumococcal clones. The second is an antibiotic-sparing indirect effect: by preventing pneumococcal disease syndromes that commonly trigger empirical antibiotic treatment, PCVs can reduce antibiotic exposure and thereby decrease selection pressure on pneumococci and other members of the nasopharyngeal microbiota (1, 3, 7). Distinguishing these two mechanisms is important because the first depends on whether vaccine serotypes overlap with resistant serotype–clone combinations, whereas the second depends on the vaccine-preventable fraction of common respiratory infections and associated prescribing behavior.

### 2.1 Direct reduction of resistant vaccine-type serotypes

The most visible AMR effect of PCVs is the selective reduction of resistant vaccine-type pneumococci. Before widespread use of the 7-valent PCV (PCV7), penicillin- and macrolide-nonsusceptible isolates were concentrated in a limited number of serotypes and serotype–clone combinations, including several PCV7 serotypes such as 6B, 9V, 14, 19F, and 23F (1, 8, 9). By reducing carriage, transmission, and invasive disease caused by these capsular types, PCV7 lowered the circulation of resistant lineages embedded within vaccine serotypes. This mechanism does not act on resistance genes directly; instead, it removes resistant clones from the population when those clones are linked to vaccine-targeted capsules.

The real-world evidence for this mechanism was established after PCV7 implementation. In the United States, PCV7 was followed by marked reductions in drug-resistant invasive pneumococcal disease (IPD), including indirect protection in unvaccinated age groups (8). However, the same period revealed the limitation of serotype-specific protection: non-PCV7 serotypes, especially serotype 19A, increased and were frequently linked to multidrug-resistant clonal backgrounds (10). PCV13 subsequently extended coverage to six additional serotypes, including 19A, and antibiotic-nonsusceptible IPD caused by the additional PCV13 serotypes declined substantially after its introduction (9, 11). The decline of macrolide-resistant serotype 19A clonal complex 320 after PCV13 further illustrates that vaccine-induced AMR reduction can occur by targeting the capsular type carried by a successful resistant clone (12). Long-term surveillance nevertheless shows that this effect remains dynamic, because reductions in vaccine-type nonsusceptible IPD can be partly offset by increases in non-vaccine-type nonsusceptible disease (13).

### 2.2 Indirect reduction of antibiotic exposure and selection pressure

PCVs may also reduce AMR indirectly by decreasing the frequency of infections for which antibiotics are commonly prescribed. Pneumococcal pneumonia, acute otitis media, sinusitis, and other acute respiratory infections are often treated empirically, especially when rapid etiological diagnosis is unavailable. Preventing a fraction of these infections can therefore reduce antibiotic consumption, which in turn reduces selective pressure on pneumococci and the broader nasopharyngeal microbiota (3, 14). This antibiotic-sparing pathway is distinct from vaccine-type suppression: it can influence AMR even when an antibiotic prescription is avoided for a syndrome that might otherwise have been treated empirically without microbiological confirmation.

The magnitude of this indirect effect is more difficult to estimate than the reduction in vaccine-type IPD. Acute otitis media provides a useful example. PCVs can reduce pneumococcal and vaccine-type acute otitis media, but the effect on all-cause acute otitis media is more modest and varies by study population, vaccine formulation, and outcome definition (15). For future vaccines, outpatient disease and antibiotic prescription endpoints may become increasingly important. A recent analysis of higher-valency PCV serotypes estimated that additional serotypes included in 15-valent and 20-valent PCVs (PCV15 and PCV20) account for pediatric outpatient visits and antibiotic prescriptions for acute respiratory infections, with PCV20-additional serotypes contributing a larger preventable burden than PCV15-additional serotypes (16). These findings support the view that pneumococcal vaccine assessment should include antibiotic-use outcomes, not only IPD and immunogenicity endpoints (5, 16).

### 2.3 Why AMR reduction remains incomplete

The direct and indirect effects of PCVs explain why vaccination can reduce pneumococcal AMR, but they also clarify why this reduction is incomplete. Direct vaccine-type suppression depends on the serotype coverage of resistant lineages; when resistant non-vaccine lineages expand, or when resistant lineages persist through non-vaccine capsular variants, AMR can remain or re-emerge. Indirect antibiotic-sparing effects also depend on vaccine uptake, the burden of vaccine-preventable respiratory disease, prescribing practices, and local antibiotic pressure. Systematic and global analyses indicate that PCV10/PCV13 implementation has reduced antimicrobial-resistant pneumococcal disease and carriage, but non-vaccine serotype replacement and residual resistant disease continue to shape the post-PCV landscape (2, 4, 13). These considerations lead to a genomic question: why can a capsular vaccine reduce AMR, yet still leave room for resistant pneumococcal lineages to persist? The answer lies in the distinct genomic organization of the capsule locus, resistance determinants, and protein vaccine targets.

## 3 Genomic architecture underlying serotype, resistance, and vaccine targets

The relationship between PCVs and pneumococcal AMR is easier to understand when the capsule, resistance determinants, and protein vaccine targets are considered as distinct genomic layers. PCVs primarily act on capsular polysaccharides, whereas antimicrobial resistance is determined by allelic variation, recombination, and mobile genetic elements distributed outside the capsule locus. In contrast, most candidate protein vaccine antigens are encoded by dispersed chromosomal genes that are not part of the capsule biosynthesis operon. This genomic separation explains why PCVs can reduce AMR at the population level without directly targeting resistance genes, and why replacement or protein-based vaccine strategies cannot be interpreted by serotype alone.

### 3.1 The capsule biosynthesis locus as the basis of serotype-specific vaccination

The pneumococcal capsule is the structural basis of serotype definition and the direct target of currently licensed PCVs. The genes required for capsular polysaccharide biosynthesis are organized at a specific chromosomal capsule locus, and variation in this locus determines the biochemical composition and antigenic specificity of the capsule. Because PCVs induce immunity against selected capsular polysaccharides, vaccine protection is inherently serotype-specific: an isolate is directly covered when its capsule is included in the vaccine formulation, but not when it expresses a non-vaccine capsule. This structure also provides the genetic basis for capsular switching. Homologous recombination can replace the capsule locus in a pre-existing genomic background, allowing a lineage that previously expressed a vaccine-type capsule to persist under a non-vaccine capsule after vaccine pressure (17–19). Therefore, the capsule locus links vaccine coverage to serotype, but it does not by itself define antimicrobial resistance or broader virulence potential.

### 3.2 Dispersed and mobile determinants of antimicrobial resistance

AMR determinants in *S. pneumoniae* are not confined to the capsule biosynthesis locus. β-lactam nonsusceptibility is largely associated with altered and mosaic penicillin-binding protein alleles generated through recombination, whereas macrolide and tetracycline resistance commonly involve mobile or mobilizable determinants such as *erm(B)*, *mef(A/E)*, and *tet(M)*, often carried on integrative and conjugative elements or related transposon-like structures (17, 18, 20). These determinants can be selected by antibiotic exposure independently of the capsule expressed by a strain. As a result, the same serotype may include both susceptible and resistant lineages, and the same resistant lineage may appear under different capsular types after recombination. Vaccine pressure and antibiotic pressure therefore act on partly different genomic substrates: PCVs reduce selected capsular types, while antibiotics select for resistance determinants within the remaining pneumococcal population (20). This distinction is essential for interpreting post-PCV AMR trends, because the decline of a vaccine-type serotype reduces AMR only when that serotype is linked to a resistant genetic background.

### 3.3 Dispersed virulence protein genes and implications for protein vaccines

The genomic organization of candidate protein vaccine antigens is different from both the capsule locus and many AMR determinants. Pneumococcal proteins considered for serotype-independent vaccines, including PspA, PsaA, Ply or detoxified Ply derivatives, PhtD, NanA, Tuf, PiuA, and other surface-exposed or virulence-associated proteins, are encoded by genes distributed across the chromosome rather than by a single serotype-defining locus (5, 19, 21–23). This dispersed organization is the rationale for protein-based vaccines: conserved antigens could, in principle, provide protection across multiple capsular serotypes and reduce dependence on ever-expanding PCV valency. However, it also creates formulation challenges. Candidate proteins must be evaluated for sequence conservation, expression across lineages, functional relevance to colonization or invasion, immune accessibility, and compatibility with existing PCV-induced immunity. Thus, future protein-based pneumococcal vaccines should not be judged only by antigen-specific immunogenicity, but also by how consistently their targets are represented across the lineages that drive carriage, disease, and AMR after PCV implementation.

## 4 Serotype replacement as lineage-level ecological restructuring

Serotype replacement is often described as an increase in non-vaccine serotypes after vaccine-type pneumococci decline. Although this description is epidemiologically useful, it is incomplete for interpreting AMR. Because capsular type, resistance determinants, and lineage background are not genetically equivalent, the AMR consequence of replacement depends on which lineages expand, persist, or switch capsules after PCV implementation. Replacement should therefore be understood as lineage-level ecological restructuring rather than a simple exchange of capsular types.

### 4.1 Why capsule alone cannot explain AMR dynamics

The same pneumococcal serotype may be expressed by multiple genetic lineages, and the same lineage may appear under different capsular types. This creates an important distinction between serotype replacement and lineage replacement. If a vaccine-type resistant lineage disappears after PCV introduction, AMR may decline. If the same lineage persists through a non-vaccine capsule, or if a resistant non-vaccine lineage expands into the ecological space left by vaccine-type strains, AMR may remain stable or increase despite successful suppression of vaccine serotypes (17, 18, 20). Thus, serotype-based surveillance is necessary for measuring PCV coverage, but it is insufficient for predicting AMR outcomes unless it is combined with lineage and resistance data.

This distinction is particularly important because pneumococci are highly recombinogenic. Vaccine pressure acts primarily on capsular types, whereas antibiotic pressure selects for resistant variants within the residual population. These pressures can converge at the population level: PCVs reduce targeted serotypes, while antibiotics favour lineages that carry resistance determinants and can survive under continuing treatment pressure. Consequently, post-PCV AMR can change through at least three routes: expansion of resistant non-vaccine lineages, persistence of resistant lineages through non-vaccine capsular variants, and emergence of capsular switch variants that retain established resistance backgrounds (17, 18, 20). The net AMR effect of PCVs therefore depends not only on which serotypes are removed, but also on which genomic lineages occupy the post-vaccine niche.

### 4.2 GPSCs and lineage-mediated replacement

The Global Pneumococcal Sequence Cluster (GPSC) framework provides a practical genomic language for interpreting this process. By grouping pneumococci into internationally comparable genomic lineages, GPSCs allow serotype, invasiveness, AMR, geographic spread, and vaccine impact to be considered within the same population structure (24). This is particularly useful because many GPSCs contain multiple serotypes, including both vaccine and non-vaccine serotypes. PCV pressure may therefore remove a vaccine-type capsular variant without eliminating the underlying lineage, leaving room for the same lineage to persist or expand under a different capsule.

An international whole-genome sequencing study of childhood invasive pneumococcal disease in the post-PCV13 era showed that replacement was mediated both by expansion of non-vaccine-type GPSCs and by expansion of non-vaccine serotypes within GPSCs that also contained vaccine-type serotypes (25). This finding directly reframes post-PCV replacement as lineage-level ecological restructuring. The same principle is illustrated by the emergence of serotype 24F after PCV13 in France and other countries, which was driven largely by a multidrug-resistant and virulent GPSC10 lineage (26). In this case, replacement became AMR-relevant not simply because a non-vaccine serotype increased, but because the expanding serotype was carried by a successful resistant lineage.

Recent genome-based studies further show that lineage fitness and AMR can be reshaped by PCV implementation in setting-specific ways. In South Africa, genomic analysis across pre-PCV, PCV7, early-PCV13, and late-PCV13 periods showed major reductions in vaccine-type lineages, but persistent disease from non-vaccine serotypes expressed by both vaccine-type and non-vaccine-type lineages; penicillin, erythromycin, and multidrug nonsusceptibility also increased among non-vaccine serotypes in the late-PCV13 period (27). A larger geographically resolved analysis of pneumococcal genomes from South Africa showed that PCV implementation altered the relative fitness of vaccine and non-vaccine types, with rising fitness of non-vaccine-type strains and evidence that early vaccine-linked decreases in AMR may not be permanent (28). Together, these studies support a shift from viewing PCV impact as serotype suppression alone to viewing it as a change in lineage fitness under combined vaccine and antibiotic selection.

### 4.3 Carriage reservoirs and longitudinal WGS evidence

Carriage is the ecological reservoir from which both invasive disease and replacement lineages emerge. Therefore, lineage-level interpretation of PCV impact should include both disease isolates and carriage populations. Longitudinal whole-genome sequencing (WGS) provides an important framework for this purpose. In Norway, a genome-based survey of invasive pneumococci over four decades showed that PCV introduction strongly shaped the disease-causing pneumococcal population, but lineage-specific responses varied. Some GPSCs initially dominated by vaccine serotypes declined, whereas others persisted through expansion of non-vaccine serotypes, illustrating how lineages can partly escape vaccine pressure through capsular diversity (29).

Studies from regions with evolving or more recent PCV uptake reinforce this dynamic view. In southern India, population genomic analysis during the early period of increased PCV use suggested that the pneumococcal population had not yet reached a stable post-vaccine equilibrium, with concurrent changes in serotype distribution, resistance determinants, and multidrug resistance (30). In Mongolia, six years after PCV13 introduction, reduced PCV13-type carriage was accompanied by non-PCV13 replacement, persistent vaccine-type carriage in some groups, and changes in AMR gene prevalence; molecular serotyping and GPSC inference provided a framework for linking carriage changes to AMR-relevant population structure (31). These studies emphasize that post-PCV AMR surveillance should not be limited to invasive isolates, because replacement begins and is maintained in the nasopharyngeal carriage reservoir.

### 4.4 Synthesis: from genomic restructuring to epidemiological impact

The lineage-centered view explains why PCV-associated AMR outcomes vary between regions. A vaccine program may reduce resistant disease when vaccine serotypes contain dominant resistant lineages, but the same program may have a smaller or more transient AMR effect if resistant non-vaccine lineages are already common, if vaccine uptake is uneven, or if antibiotic pressure remains high. Therefore, evaluating PCV impact on AMR requires integration of serotype distribution, GPSC or other genomic lineage assignment, resistance determinants, carriage dynamics, and local antibiotic use. This provides the conceptual bridge to global and regional epidemiology: lineage-level restructuring is the genomic mechanism, whereas regional differences in serotype distribution, pathogenic capacity, vaccine uptake, and antibiotic pressure determine the observable public health impact.

## 5 Global and regional epidemiological impact of PCVs on AMR

The genomic processes described above are expressed epidemiologically as changes in disease incidence, serotype distribution, antibiotic nonsusceptibility, and age- or region-specific disease burden. Therefore, the impact of PCVs on AMR should be interpreted at both global and regional levels. Globally, PCV10 and PCV13 have substantially reduced IPD, especially in young children, and have also contributed to reductions in antimicrobial-resistant pneumococcal disease and carriage when vaccine serotypes contained dominant resistant lineages (2, 4, 7). However, these benefits have occurred alongside expansion of non-vaccine serotypes, meaning that the long-term AMR effect of PCVs depends on the resistance profiles, lineage backgrounds, and invasive potential of the replacing population.

### 5.1 Global impact and non-vaccine serotype expansion

Global and systematic analyses show that the public health impact of PCVs is large but not static. The PSERENADE project demonstrated that long-term PCV10 or PCV13 use substantially reduced IPD in children and produced more moderate indirect effects in older age groups, but non-vaccine-type disease increased over time after vaccine introduction (4). Similarly, a systematic review focused on antimicrobial-resistant pneumococcal disease and carriage found reductions in resistant vaccine-type pneumococci after PCV10/13 introduction, while emphasizing that post-vaccine replacement can modify the net AMR effect (2). A global systematic review and meta-regression of pediatric pneumococcal isolates also support the view that PCV implementation has altered AMR patterns, particularly where resistant disease was concentrated in vaccine-covered serotypes (7). These findings indicate that PCVs can reduce AMR at the population level, but this reduction should be interpreted as a dynamic ecological effect rather than a permanent elimination of resistant pneumococci.

### 5.2 Serotype-specific pathogenic capacity and geographic distribution

The epidemiological consequence of serotype replacement depends not only on whether a replacing serotype is vaccine-covered, but also on its capacity to cause disease. Pneumococcal serotypes differ in capsule composition, carriage prevalence, duration of colonization, invasiveness, age distribution, and geographic distribution. A global meta-analysis of paired carriage prevalence and IPD incidence showed that serotype-specific invasiveness, estimated using case-carrier ratios, varies by serotype, age group, income setting, human immunodeficiency virus (HIV) status, and pre- versus post-PCV period (32). This means that replacement by a highly carried but weakly invasive serotype may have a different public health consequence from replacement by a less common but highly invasive serotype. Regional serotype composition is also important for vaccine policy. In Europe, post-PCV10/13 replacement has involved multiple non-PCV13 serotypes, whereas systematic review of PCV20-additional serotypes highlights the epidemiological relevance of serotypes such as 8, 10A, 11A, 12F, 15B/C, 22F, and 33F in the post-PCV13 era (33, 34). Thus, global vaccine assessment should not treat all non-vaccine serotypes as equivalent; their pathogenic potential and regional distribution determine whether replacement becomes clinically and AMR-relevant.

### 5.3 Regional heterogeneity: mature PCV programs versus uneven uptake settings

Regional heterogeneity is central to interpreting PCV-associated AMR outcomes. In countries with mature childhood PCV programs and strong surveillance systems, the dominant problem is often residual disease caused by non-vaccine serotypes, together with the emergence or persistence of resistant non-vaccine lineages. A 10-country European study showed that IPD incidence declined after PCV10/13 introduction but later increased in association with non-PCV13 serotype replacement, illustrating how the benefits of vaccine-type suppression can be partially offset by non-vaccine disease (33). In contrast, regions with lower, delayed, or uneven PCV uptake may continue to show a high burden of vaccine-type or PCV13-covered serotypes, limiting the observable AMR benefit of PCVs. This situation is particularly relevant to China, where PCV13 has long been available mainly as a self-funded vaccine rather than as a uniformly implemented national immunization program. In Southwest China, whole-genome characterization of isolates from all age groups during 2018–2022 showed that 19F, 19A, 3, 23F, 6A, and 23A remained common, with prevalent sequence types including ST271 and ST320 and a high burden of macrolide resistance and multidrug resistance (35). These data emphasize that PCV-driven AMR reduction is regional and conditional: it depends on baseline serotype-lineage composition, vaccine uptake, antibiotic pressure, and the capacity of local lineages to persist or expand after vaccine implementation.

Together, the global and regional evidence supports an integrated interpretation of PCV impact on AMR. PCVs can reduce resistant pneumococcal disease when they remove dominant resistant vaccine-type lineages and lower antibiotic exposure, but replacement outcomes depend on the serotype-specific invasiveness and geographic distribution of the residual population. Therefore, AMR-oriented vaccine policy should combine serotype surveillance with lineage assignment, resistance profiling, carriage studies, antibiotic-use data, and region-specific assessment of vaccine coverage. **Table 1** summarizes the major evidence layers linking PCV mechanisms, serotype-lineage dynamics, AMR interpretation, and future vaccine assessment. This framework provides the basis for evaluating higher-valency PCVs, protein-based vaccines, and host-directed vaccine strategies in the next section.

**Table 1:** Representative evidence linking PCV mechanisms, serotype-lineage dynamics, antimicrobial resistance, and future pneumococcal vaccine assessment.

Evidence layer/vaccine era	Representative evidence	Serotype–lineage dynamics relevant to AMR	AMR consequence/interpretation	Implication for future vaccine assessment
PCV7 era: vaccine-type resistant reservoirs (1, 8, 10)	Pre-PCV resistant disease was concentrated in several vaccine-type serotypes; PCV7 introduction reduced drug-resistant IPD, while non-PCV7 disease, notably 19A, increased.	PCV7 suppressed major resistant vaccine-type serotype–clone combinations, but left ecological space for resistant non-vaccine clones.	Vaccination reduced resistant disease through a direct population-level effect on targeted capsular reservoirs, not by molecularly targeting resistance genes.	Vaccine impact studies should link serotype decline to resistant clone decline, not only to overall IPD incidence.
PCV13 era: correction of 19A-associated AMR (11–13)	PCV13 added 19A and other serotypes; antibiotic-nonsusceptible IPD due to added PCV13 serotypes declined, including macrolide-resistant 19A CC320.	A successful resistant serotype–clone combination was targeted, but residual disease shifted toward non-vaccine serotypes.	Expanded coverage corrected a major replacement-associated AMR problem, but residual nonsusceptible disease remained dynamic.	Higher-valency design should prioritize serotypes carried by successful resistant lineages.
Global and systematic PCV10/13 evidence (2, 4)	Systematic and global analyses show that PCV10/13 reduced pneumococcal disease and AMR-related outcomes, while non-vaccine serotypes increased over time.	Replacement magnitude and dominant serotypes vary by setting, vaccine formulation, and baseline population structure.	The net AMR effect is context-dependent rather than a simple linear decline.	Population-level assessments should include AMR and replacement endpoints alongside IPD reduction.
Genomic interpretation: replacement as lineage restructuring (17, 18, 24, 25)	Population-genomic studies showed rapid pneumococcal adaptation and defined GPSCs to link serotype, lineage, resistance, and vaccine impact.	The same serotype can occur in multiple lineages, and the same lineage can persist under different capsules.	Serotype-only surveillance may miss whether replacement introduces susceptible, resistant, or high-fitness lineages.	Post-PCV surveillance should integrate serotype, MLST/GPSC, resistance determinants, and capsular switching.
Recent WGS evidence: lineage-driven AMR replacement (26–29)	Post-PCV WGS identified MDR/virulent lineage-mediated replacement, including 24F/GPSC10, and quantified PCV-associated fitness shifts.	Non-vaccine lineages can expand across regions; vaccine pressure and antibiotic selection jointly shape lineage fitness.	Replacement becomes AMR-relevant when expanding lineages are MDR, virulent, or antibiotic-selected.	Candidate vaccines should be assessed by resistant lineage coverage and predicted residual population structure.
Regional surveillance: heterogeneous post-PCV outcomes (29–31, 33)	European multi-country and regional WGS studies show that local baseline lineages, vaccine uptake, and antibiotic pressure shape post-PCV outcomes.	Mature PCV programs often show residual non-vaccine disease; lower or uneven uptake may leave vaccine-type lineages prominent.	PCV-driven AMR reduction is regional and conditional, not universal.	Regional policy should be guided by local serotype–lineage–AMR surveillance.
Higher-valency PCVs and PCV24 (36, 37, 39, 40)	PCV15/20/21 broaden serotype coverage; VAX-24 shows feasibility by immunogenicity and safety, but lacks real-world AMR evidence.	Added serotypes may capture current resistant disease, but broader valency still remains vulnerable to future replacement.	AMR impact depends on coverage of resistant serotype–lineage combinations, not valency alone.	Higher-valency PCVs should be evaluated with carriage, AMR, and lineage endpoints, not immunogenicity alone.
Beyond valency: protein and trained-immunity-based strategies (5, 41, 43, 48, 50)	Future strategies include conserved-antigen vaccines and host-directed trained-immunity concepts; experimental data link trained innate immunity to pneumococcal protection.	Protein vaccines target conserved antigens; trained-immunity approaches aim to reprogram host innate responses rather than target serotypes.	Direct PCV-trained-immunity evidence is limited, but pneumococcal host defence can be modulated by trained innate immunity.	Next-generation vaccines should be assessed by infection reduction, antibiotic exposure, carriage, lineage expansion, and immune endpoints beyond antibody titres.

AMR, antimicrobial resistance; CC, clonal complex; GPSC, Global Pneumococcal Sequence Cluster; IPD, invasive pneumococcal disease; MDR, multidrug-resistant; MLST, multilocus sequence typing; PCV, pneumococcal conjugate vaccine; PCV7/10/13/15/20/21/24, 7-/10-/13-/15-/20-/21-/24-valent pneumococcal conjugate vaccine; VAX-24, a 24-valent pneumococcal conjugate vaccine candidate; WGS, whole-genome sequencing.

## 6 Future vaccine formulation and AMR-oriented assessment

The PCV experience shows that vaccine formulation should not be evaluated only by the number of serotypes included or by short-term immunogenicity. For AMR control, the key question is whether a vaccine can reduce disease, carriage, antibiotic exposure, and transmission of resistant pneumococcal lineages without rapidly creating ecological space for replacement by other AMR-associated populations. Future pneumococcal vaccine assessment should therefore integrate serotype coverage, resistant lineage coverage, regional epidemiology, protein antigen conservation, and host-directed immune endpoints.

### 6.1 Higher-valency PCVs: broader coverage with ecological constraints

Higher-valency PCVs are the most immediate response to post-PCV13 replacement. The 15-, 20-, and 21-valent PCVs (PCV15, PCV20, and PCV21) expand capsular coverage and may reduce additional disease and AMR burden when their added serotypes overlap with locally prevalent resistant serotype-lineage combinations (34, 36–39). Recent AMR-focused analysis from Belgium suggested that the potential effect of higher-valency PCVs on antimicrobial resistance rates depends on whether additional vaccine serotypes are major contributors to resistant invasive disease in that setting (36). Similarly, PCV20 and PCV21 recommendations illustrate how vaccine policy is increasingly shaped by residual adult disease and serotype distribution rather than by childhood PCV13-era assumptions alone (37–39). VAX-24 further demonstrates the feasibility of broader-valency conjugate vaccine design, but current evidence remains primarily based on safety and immunogenicity rather than long-term effects on carriage, antibiotic use, replacement, or AMR ecology (40). Thus, higher valency can narrow existing coverage gaps, but it does not remove the need for genomic and epidemiological surveillance.

### 6.2 Protein-based vaccines: criteria beyond antigen conservation

Protein-based pneumococcal vaccines provide a complementary strategy because they aim to reduce dependence on capsular serotype coverage. Candidate antigens such as PspA, PsaA, Ply or detoxified Ply derivatives, PhtD, NanA, Tuf, PiuA, and other surface-exposed or virulence-associated proteins are attractive because they may be conserved across multiple serotypes and could, in principle, provide broader protection than capsule-based formulations (5, 19, 21–23). However, antigen conservation alone is not sufficient for vaccine formulation. Candidate proteins should also be assessed for broad expression across major lineages, surface exposure or release during infection, functional relevance to colonization or invasion, immunogenicity in both mucosal and systemic compartments, and ability to reduce carriage or transmission. These criteria are particularly important for AMR-oriented vaccine development, because a protein vaccine that reduces disease but has little effect on carriage may have limited impact on resistant lineage circulation. Conversely, a vaccine that reduces colonization or transmission across multiple lineages could complement PCVs by limiting the reservoir from which resistant replacement populations emerge. Therefore, protein-based vaccine candidates should be evaluated not only as immunogens, but also as ecological interventions that may alter pneumococcal population structure.

### 6.3 Trained-immunity-based and host-directed strategies

Trained-immunity-based vaccines represent a distinct host-directed concept rather than an extension of PCV biology. Trained immunity is characterized by long-term functional reprogramming of innate immune cells through epigenetic and metabolic mechanisms, leading to altered responses to subsequent infectious challenges (41). Sánchez-Ramón and colleagues proposed trained-immunity-based vaccines as broad-spectrum anti-infective formulations, and later work has further developed this concept for infection prevention and vaccine design (6, 42, 43). In the respiratory infection field, mucosal polybacterial formulations such as MV130 provide clinically oriented examples: they have been associated with reduced recurrent infections and antibiotic use in selected patient groups, and experimental work supports trained-immunity-associated immune reprogramming (44–47). For pneumococcal disease specifically, experimental studies show that Bacille Calmette-Guérin (BCG)-induced or locally induced trained innate immunity can enhance protection against pulmonary pneumococcal challenge, while pneumococcal endopeptidase O has been reported to induce trained immunity and confer broad protection against multiple pathogenic infections (48–50). These findings do not imply that licensed PCVs act through trained immunity, but they support the broader concept that host-directed immune training may complement antigen-specific vaccine strategies.

### 6.4 AMR-oriented endpoints for next-generation vaccines

Future pneumococcal vaccine studies should incorporate AMR-oriented endpoints from the outset. For higher-valency PCVs, protein vaccines, and host-directed strategies, evaluation should include vaccine-type and non-vaccine-type carriage, carriage density and duration, antibiotic prescriptions, resistance determinants, resistant lineage expansion, capsular switching, and region-specific population-genomic effects (3, 5, 16). Immune-monitoring strategies should also extend beyond serotype-specific antibody titres when host-directed or protein-based vaccines are considered. Simplified approaches to cellular immune assessment, such as delayed-type hypersensitivity-based monitoring in vaccine studies, illustrate one possible direction for more accessible immune readouts, although such approaches require validation in pneumococcal vaccine contexts (51). Ultimately, next-generation pneumococcal vaccines should be judged not only by breadth of immune recognition, but also by their ability to reduce infection burden, limit antibiotic exposure, and avoid creating ecological space for new resistant lineages.

## 7 Limitations of this mini review

This Mini Review has several limitations. First, it was designed as a narrative Mini Review rather than a systematic review or meta-analysis. Therefore, we did not perform a formal systematic search using predefined search strings, risk-of-bias assessment, study-quality grading, or quantitative evidence synthesis. Instead, we selected representative peer-reviewed and PubMed-indexed studies that were directly relevant to PCVs, pneumococcal AMR, serotype replacement, population genomics, higher-valency vaccines, protein-based vaccine strategies, and trained-immunity-based approaches. We also prioritized recent studies and high-impact evidence where available, while retaining selected classic studies that established the relationship between PCV implementation and changes in drug-resistant pneumococcal disease.

Second, the evidence summarized in this review is heterogeneous. Studies differ in vaccine formulation, dosing schedule, age group, vaccine coverage, surveillance intensity, pneumococcal sampling strategy, disease endpoint, antimicrobial susceptibility testing method, breakpoint interpretation, and availability of antibiotic-use data. In addition, many AMR-focused studies rely mainly on invasive disease isolates, whereas carriage, acute otitis media, non-invasive pneumonia, outpatient antibiotic prescribing, and longitudinal genomic surveillance are less consistently captured across regions (2, 4, 7). These differences limit direct comparison between settings and may partly explain why the apparent AMR impact of PCVs varies across countries and time periods.

Third, trained-immunity-based strategies are discussed here as a future and complementary direction, not as an established mechanism of licensed PCVs. Although trained immunity is now well defined immunologically, and selected experimental studies support trained innate protection against pneumococcal challenge or pneumococcal protein-induced immune training, direct evidence linking PCVs themselves to trained immunity remains limited (41, 50). Accordingly, this review treats trained immunity as a host-directed perspective for future vaccine development rather than as a proven explanation for current PCV-mediated AMR effects.

## 8 Conclusions

PCVs provide a well-established example of how vaccination can reshape bacterial AMR at the population level. Their AMR impact is mediated through two complementary pathways: direct reduction of vaccine-type serotypes that historically carried resistant lineages, and indirect reduction of antibiotic exposure by preventing pneumococcal and respiratory disease syndromes that commonly lead to empirical treatment. However, this effect is not fixed or universal. Because the capsule locus, AMR determinants, and virulence protein genes represent distinct genomic layers, the long-term outcome of PCV implementation depends on serotype replacement, capsular switching, lineage fitness, antibiotic pressure, and regional vaccine uptake.

Future pneumococcal vaccine formulation should therefore move beyond simple serotype counting. Higher-valency PCVs should be assessed according to their coverage of locally important resistant serotype-lineage combinations, while protein-based vaccines require evaluation of antigen conservation, expression across lineages, immune accessibility, and potential effects on carriage and transmission. Host-directed strategies, including trained-immunity-based approaches, may provide complementary opportunities but should be evaluated with population-level AMR endpoints rather than immunological readouts alone. Overall, next-generation pneumococcal vaccines should be judged by their ability to reduce disease burden, limit antibiotic use, suppress resistant lineage expansion, and avoid creating new ecological niches for AMR-associated pneumococci.
